# Spit It Out West Virginia: a perspective of smokeless tobacco cessation and education in rural West Virginia

**DOI:** 10.3389/fpubh.2025.1503858

**Published:** 2025-02-26

**Authors:** Donald Reed, Stephanie Lusk, Truman Dangerfield, Mallory Ball, Janna Stoner, Michele Bowles

**Affiliations:** ^1^School of Health Sciences, Liberty University, Lynchburg, VA, United States; ^2^McDowell County Commission on Aging, Welch, WV, United States; ^3^Marriott School of Business, Brigham Young University, Provo, UT, United States; ^4^Pallottine Foundation, Huntington, WV, United States; ^5^West Virginia Division of Tobacco Prevention, Charleston, WV, United States

**Keywords:** smokeless tobacco, cessation, workshop, community, rural

## Abstract

This article highlights strategies for implementing successful rural public health campaigns by sharing insights from the *Spit It Out – West Virginia* Project. Active since 2007, the project expanded to Mingo County in 2023 to address high smokeless tobacco use through culturally relevant education and cessation workshops. Key components included leveraging local partnerships, tailoring campaigns to community norms, and integrating accessible support services like the West Virginia Tobacco Quitline. Outcomes demonstrated increased awareness, behavioral change, and community engagement. Our perspective offers practical lessons for designing effective, community-based interventions in rural settings to address pressing public health challenges.

## 1 Introduction

Smokeless tobacco is sucked or chewed instead of being burned like traditional cigarettes. There are many types of smokeless tobacco products. These include chewing tobacco (cured tobacco that is chewed and then spit), dry snuff (finely cut or powdered tobacco sniffed through the nostrils), snus (cut tobacco that is pouched, or loose in the mouth), and dissolvable tobacco (lozenges, sticks, strips or hard candy that are not spit) (1, 2). One common ingredient in these tobacco products is nicotine. This chemical is responsible for the addictive quality of smokeless tobacco and other tobacco products. In addition to its addictive nature, there are 28 known carcinogens in smokeless tobacco (3). These carcinogens and other ingredients make smokeless tobacco associated with both short and long-term health problems. Some of the long-term health problems include nicotine addiction and nicotine poisoning in children (4). Smokeless tobacco has also been shown to increase oral diseases, cardiovascular disease, and risks for early delivery and stillbirth. Smokeless tobacco causes white or gray patches in the mouth (leukoplakia) which can eventually lead to mouth cancer. It has also been shown to increase the risk of other types of cancer including cancer of the esophagus and pancreas.

The prevalence of smokeless tobacco use among adults in the United States is 2.1% (5). In West Virginia, the rate is much higher, with a stark contrast between rates for men at 15.7% and women at 1%, making a combined rate of 8.2% of the adult population (6). This makes the smokeless tobacco use rate in West Virginia from 2 to 4 times the national average and the highest in the nation (7, 8).

Currently, many state tobacco control programs are heavily focused on addressing vaping and other electronic nicotine delivery systems. However, there is a significant risk that smokeless tobacco use will rise in rural states as cigarette use declines and efforts to combat vaping intensify. It is crucial for West Virginia and these other states to prioritize smokeless tobacco prevention education and cessation services to address this growing concern.

## 2 Cultural context

Another reason West Virginia has such a high smokeless tobacco use rate is found in the cultural context of the state. Since the late 1800s, West Virginia has been a coal mining state. Despite decreasing its production in recent years, this state is still a large coal producer, and many of the people who live in West Virginia are employed as coal miners (9). In 2022, 10,219 people worked in underground mines (10). Smoking was prohibited inside underground mines because of safety concerns by the Federal Mine Safety and Health Act of 1977 (11). This regulation has led many mine workers to use smokeless tobacco, creating a strong cultural preference for it. A 2014 report published by the Centers for Disease Control and Prevention found that 18.8% of those in mining industries use smokeless tobacco (12, 13).

Rural communities are uniquely and adversely affected by their low population density and reduced access to essential social and economic resources (14). In rural Appalachia, smokeless tobacco is perceived as a rite of passage, a sign of masculinity, and a key to social acceptance. Tobacco marketing loopholes, the low cost of smokeless tobacco, poverty, stress, and a reluctance to implement effective tobacco control policies, all of which are common characteristics among rural populations, has led to a cultural acceptance of smokeless tobacco use (15).

## 3 Expansion of successful model

The “Spit It Out – West Virginia” Project, supported by the West Virginia Division of Tobacco Prevention, the TRUTH Initiative, and the Pallottine Foundation of Huntington, began in 2007 and remains active. Recognized as a Rural Health Model Case Study by the Rural Health Information Hub, the project expanded to Mingo County in June 2023 to address smokeless tobacco use. It conducted five tobacco education workshops and five cessation workshops, each targeting 60 participants, with no overlap between the groups. Education workshops focused on the health impacts of smokeless tobacco and aimed at youth, parents, and grandparents to encourage information-sharing within families. Cessation workshops provided strategies for quitting and targeted current users. Pre- and post-surveys were collected for both types of workshops.

Workshops were held in community settings such as fire departments, senior centers, and coal mining industries. A registered nurse offered voluntary oral health screenings at each event, and cessation workshop participants received follow-up calls after 30 days to assess tobacco use and Quitline engagement.

The project also promoted *Through With Chew Week* (February 19–23, 2024), with an emphasis on the *Great American Spit Out* campaign. Advertisements and social media outreach targeted blue-collar men and generational smokeless tobacco use, featuring local figures such as Dr. Susan Morgan, Greg Puckett, and Cynthia Keely. These campaigns, highlighting the health risks of tobacco, generated 27,339 impressions.

## 4 Program evaluation results

The project ran for about one year, from June 1st, 2023, to May 30th, 2024. Despite its short timeframe, the Spit It Out West Virginia project impacted hundreds of people and helped increase understanding of the dangers of tobacco use and knowledge of the resources available to assist in quitting.

Twenty-two Mingo County organizations were initially contacted via mail with information about the Spit It Out West Virginia project. All these organizations received follow up contact via telephone to gauge interest in hosting a workshop. Eight educational workshops were held at seven community organizations and five cessation workshops were held at three community organizations. Additionally, an educational tobacco prevention booth was set up at three community events and reached 353 people. These community events included two health fairs and one Trailfest for UTV enthusiasts.

The workshops were held at a variety of community organizations. For example, educational workshops were held at the Delbarton Town Hall, the Mingo County Sheriff’s Department, Veteran’s Services, the Chatteroy Church of God, and the Coalfields Community Action Partnership Senior Centers in Matewan and Gilbert. The cessation workshops were held at the Williamson Fire Department, Williamson Community Smoking Cessation, and Serenity Pointe, a sober living facility. In total, the eight educational workshops had 60 participants, and the five cessation workshops had 56 participants.

Through the surveys provided to the participants, we measured the changes in self-reported attitudes toward and understanding of tobacco use using a weighted Likert scale. After the educational workshop, there was a 65.80% increase in the response to the statement, “I can explain the WV Tobacco Quitline,” as well as a 34.87% increase in the response to the statement, “I understand marketing strategies used in smokeless tobacco advertising” (Table 1). This increased understanding of the Quitline makes the participants better able to assist friends and family who use smokeless tobacco. After the cessation workshop, there was a 25.71% increase in the response to the statement, “I know how to deal with the stress of quitting tobacco,” as well as a 20.60% increase in the response to the statement, “I know about medicines that can help me quit” (Table 2). This increased understanding of the medicines that aid people in quitting is essential to help participants feel motivated to quit.

**Table 1:** Shows the survey responses to the questions that were posed to the participants of the educational workshops.

Question	Pre-training survey	Post-training survey	Change
I understand the chemicals in spit tobacco.	3.68	4.58	24.46%
I understand nicotine addiction.	3.89	4.61	18.51%
I understand the health effects of spit tobacco use.	3.84	4.63	20.57%
I understand the financial cost of daily tobacco use.	4.07	4.68	14.99%
I understand marketing strategies used in smokeless tobacco advertising.	3.47	4.68	34.87%
I can explain the WV Tobacco Quitline.	2.69	4.46	65.80%

It was conducted using a weighted Likert scale. It highlights the pre-training responses, the post-training responses, and the percent change. *N* = 57.

**Table 2:** Shows the survey responses to the questions that were posed to the participants of the cessation workshops.

Question	Pre-training survey	Post-training survey	Change
I want to quit using tobacco.	3.25	3.32	2.11%
I understand nicotine addiction	4.06	4.08	0.49%
I understand the steps to quitting	3.60	4.02	11.67%
I understand how the body responds to nicotine withdrawal.	3.65	3.9	6.85%
I know about medicines that can help me quit.	3.35	4.04	20.60%
I am confident I can stop using tobacco.	3.19	3.38	5.96%
I am confident I can control my urges to use tobacco.	3.22	3.35	4.04%
I know how to deal with the stress of quitting tobacco.	2.8	3.52	25.71%
My friends and family will support my efforts to quit.	3.8	3.91	2.89%

It was conducted using a weighted Likert scale. It highlights the pre-training responses, the post-training responses, and the percent change. *N* = 51.

One month after the cessation workshop, any participant who provided contact information was contacted by phone, email, or mail. Of the 56 participants in the cessation program, 11 responded, for a 19.6% response rate. Information collected from this short survey assessed if the participant intended to quit using tobacco, had made a quit plan, or utilized the services from the West Virginia Tobacco Quitline. One of the 11 respondents called the tobacco Quitline. Eighteen percent of respondents (*n* = 10) reported that they quit using tobacco within the last 6 months. Seventy-five percent of respondents reported that they wished to quit using tobacco in the next 6 months. Incredibly, 100% of respondents reported that they had been able to reduce the amount of tobacco they use since attending the workshop.

Information on the West Virginia Tobacco Quitline was shared with participants in both the educational and cessation classes. This information was also shared during community health events. Enrollment for the Mingo County Quitline from June 2023 to May 2024 totaled 103 participants. This was a 30% increase from the year before the project.

Oral health screenings were offered, but not required, at every workshop. Seventeen of 116 (14.7%) participants received an oral health screening. During screenings, the head, neck, and mouth were inspected and palpated for abnormal findings. Those without a current dentist were encouraged to find a dentist in their area and schedule an appointment. While several participants had signs of gum disease, no lesions were present during exams.

## 5 Discussion on perspective of practical implications for rural tobacco control work

Tobacco education and cessation is best addressed at the local level. West Virginia has county-level coalitions that are funded by West Virginia Bureau for Behavior Health and the Federal Drug Free Communities Support Program. These county level coalitions are supported by the state-level, Coalition for Tobacco Free West Virginia, which is funded by the West Virginia Division of Tobacco Prevention. These coalitions endeavor to follow Bronfenbrenner’s ecological systems theory, while engaging community leaders to implement the Centers for Disease Control’s (CDC) Best Practices in Tobacco Control recommendations at the county level (16).

Essential to the success of this project was recruiting and engaging various community organizations. These community organizations were a vital component to the project because without their participation, it would have been difficult, if not impossible to host the workshops. The process to recruit participation of community organizations consisted of first, identifying target organizations and sending them the relevant information by mail. The informational mailings were subsequently followed by telephone calls to discuss the workshops in more detail. This dual method of communication not only made it easier to involve community organizations, but it also increased the potential for them to participate. Recruitment of individual participation consisted of a similar process. The volunteers used the Jehovah Witness Pioneer Mapping Method (without proselytizing) to contact community members.

To motivate individuals to participate in the cessation workshops, gift cards were offered to participants. Although the cessation classes were local, they still required an investment of time. The gift cards helped people be willing to leave their other responsibilities and attend the workshop. While oral cancer screenings are quick, painless, and relatively non-intrusive, they can still be uncomfortable. Offering gift cards increased individual willingness to participate in the screening.

The Through with Chew Week campaign was not a generalized campaign; instead, it incorporated West Virginia cultural themes that related to smokeless tobacco use. Through employing a campaign with cultural influence, the messages were more relevant to the target audience and had a greater impact. By leveraging these cultural themes and making them relatable, such as a passion for hunting, the campaign spoke to its audience and therefore was more effective at changing attitudes about smokeless tobacco use.

The workshops described in this project were effective because they followed up with participants. While there may have been a low response to the follow-up survey, those who did reported positive outcomes. Additionally, the constant availability of the West Virginia Tobacco Quitline provides an accessible resource to participants, their friends, and their family. The 30% increase in enrollments provides evidence that the workshops and follow-up helped people feel motivated to call. We did not follow up with participants in the education workshops, only the cessation workshops – and we realize that this was an error – we should have followed up to measure what the education workshop participants did with the knowledge gained. We did not follow up with the education workshops because our outcome measurement was mainly focused on cessation.

## 6 Limitations

### 6.1 Data collection

Tobacco use data were derived from self-reported surveys and statistical estimates, which may be affected by reporting biases and inaccuracies.

### 6.2 Generalizability

This evaluation focused on Mingo County, WV. While many practices and lessons may apply elsewhere, the county’s unique economic, cultural, and regulatory environment could limit broader applicability. Community-based intervention effectiveness may vary by region.

### 6.3 Program impact measurement

Isolating the intervention’s impact from other factors is challenging. Longer, more rigorous studies may be needed to assess the program’s full effects.

### 6.4 Short-term scope

The year-long *Spit It Out West Virginia* project provides short-term results. Extended implementation could better evaluate long-term outcomes of the educational and cessation workshops.

## Data Availability

The original contributions presented in the study are included in the article/supplementary material, further inquiries can be directed to the corresponding author.
